# Finite Element Evaluation of the Effect of Adhesive Creams on the Stress State of Dentures and Oral Mucosa

**DOI:** 10.1155/2021/5533770

**Published:** 2021-05-08

**Authors:** Anantha Narayanan Ramakrishnan, Oliver Röhrle, Christopher Ludtka, Roshan Varghese, Josephine Koehler, Andreas Kiesow, Stefan Schwan

**Affiliations:** ^1^Fraunhofer Institute for Microstructure of Materials and Systems IMWS, Department of Biological and Macromolecular Materials, Halle, Germany; ^2^University of Stuttgart, Institute for Modelling and Simulation of Biomedical Systems, Faculty of Civil and Environmental Engineering, Pfaffenwaldring 5a, Stuttgart, Germany; ^3^University of Florida, J. Crayton Pruitt Family Department of Biomedical Engineering, 1275 Center Drive, Biomedical Sciences Building JG56 P.O. Box 116131 Gainesville, FL 32611-6131, USA; ^4^GSK, Denture Care & Dry Mouth, St Georges Ave, Weybridge, Surrey KT13 0DE, UK; ^5^Martin-Luther-University Halle-Wittenberg, Department of Prosthodontics, School of Dental Medicine, Magdeburger Str., 16 Halle, Germany

## Abstract

The base fit between a removable partial denture (RPD) and the underlying soft tissue plays a significant role in its performance. The application of a denture adhesive is hypothesized to result in better retention of RPDs and, as a result, contribute to lower stress on the oral mucosa. The objectives of this study were to observe and compare the distribution of simulated bite forces applied to the RPD through the abutments and soft tissue for models with and without the use of a denture adhesive. Furthermore, we evaluated the possible benefit of using a denture adhesive in lowering stresses on the oral mucosa. The RPD, mandible, oral mucosa, abutment teeth supporting the RPD, and the corresponding abutment periodontal ligaments (PDLs) were modelled as 3D volumes based on computer tomography (CT) datasets. A viscoelastic adhesive layer between the RPD and oral mucosa was incorporated into this base model using Prony series approximation. The layer was developed as a volume extract using the denture surface. Finite element (FE) simulations were performed for the bite force on one of the RPD segments, with the resulting force and moments experienced by the dental structures and oral mucosa compared between the model with the adhesive layer and the base model without. As a result, the contact pressure on the oral mucosa for the model with the denture adhesive decreased to 0.15 MPa as compared to 0.25 MPa for the model without the adhesive. The potential role of denture adhesives in leading to a better fit between the RPD and oral mucosa as well as lowering contact pressures could be used to improve comfort in patients wearing RPDs.

## 1. Introduction

A dental cast is any dental prosthetic or device formed in mold or used as a mold. They serve to replicate a patient's teeth and oral structures for diagnostic purposes and are used as models for further casting of dental prosthetics such as bridges, crowns, implants, dentures, and partial dentures produced based on the negative replica of the patient's teeth. Removable partial dentures (RPDs) or implants cast in this manner fit into the patient's jaw structure and resemble the other teeth surrounding them. In the case of partial dentures cast for the mandible, or lower jaw, the dentures are designed to rest on the surrounding teeth, known as abutment teeth as well as directly on the soft tissue. Specially designed clasps are provided at the contact spaces between the denture and the abutment teeth. These clasps rest either on the top occlusal surface, the lingual and labial faces of the abutment teeth, or a combination of both. The bite force applied to the dentures is transmitted to the abutments through these clasps. The motion of the denture in the occlusal direction is also resisted to a limited extent by these clasps as well as by the underlying bone surface. However, the occlusal loads tend to be higher than the retentive force generated by the clasps [1].

Partial dentures are also in direct contact with the soft tissue, i.e., the oral mucosa, and through it, to the underlying jawbone. The base fit or retention between the partial denture and the oral mucosa plays a significant role in its performance. RPD design principles, such as appropriate base fit [2], can help preserve periodontal health. The share of the bite force distributed through the oral mucosa influences the retention and therefore the base fit. The force distribution between the elements of a system depends on the relative stiffness of each member [3]. Both vertical and horizontal forces as well as eccentric loading acting on dental restorations can induce bending moments which are assumed to generate high strains at the implant-bone interfaces [4].

Denture adhesives can play a vital role in ensuring an appropriate base fit between the RPD and oral mucosa. The adhesive creates a layer between the denture and oral mucosa that restrains movement of the denture. The desired function of the adhesive is to decrease lateral and vertical movement of the dentures and to increase incisal bite force [5]. The proper use of denture adhesives is beneficial to the patient for increasing retention and stability [6]. Most adhesives exhibit viscoelastic behavior, especially at higher stress levels [7], as such, a suitable mathematical model to ensure time-dependent behavior is needed to evaluate such materials. The most popular mathematical form to describe such viscoelastic behavior is given by the Prony series approach [8]. Finite element (FE) simulation is utilized in this study to visualize the role played by a denture adhesive in the distribution of bite forces, as an experimental evaluation of the detailed influences of adhesives is difficult to achieve. The objectives of the current study were (i) to observe and compare the distribution of bite forces through the abutments and soft tissue for the model with and without the use of denture adhesive during the application of occlusal force on the RPD and (ii) to evaluate the possible benefit of using a denture adhesive in lowering the stresses on the oral mucosa.

## 2. Methods

A representative anatomical model (SAWBONES EUROPE AB, Sweden, 216 16 Malmö, SKU: 1338-9) was used to develop computer tomography (CT) data sets and the resulting 3D volume stacks for the mandible, oral mucosa, and dental structures. The three abutment teeth in the model were the right and left canine and the 2^nd^ molar. Segmentation and segregation of the CT data sets were carried out using the software tool AMIRA. The 3D volume stacks were assembled, and surface geometries were developed using the software tool CATIA for the individual components, i.e., the mandible, the oral mucosa, the three abutment teeth, the partial denture, and the denture connector with the denture clasps resting on the three abutment teeth. This assembly was referred to as the base model, i.e., the model without the adhesive. The direct contact between the denture and oral mucosa in the base model was modelled as a frictional contact formulation with a coefficient of 0.3 based on literature sources [9, 10]. The bonded contact formulation was avoided, as it would restrain the relative displacements of the partial denture and underestimate the effect of poor retention on the soft tissue and other contact spaces.

**Figure 1:**
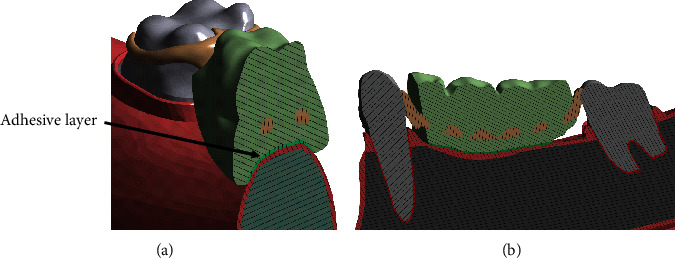


The adhesive was generated in this base 3D model by using the contact surface between the partial denture and the oral mucosa. A uniform solid layer of 0.3 mm thickness was generated from the surface extrapolated based on the current geometry and discussion in the literature on the thickness of adhesives [5, 11]. The resulting model with the adhesive layer was referred as the “adhesive model.” The adhesive introduced two new contact spaces in the model: the contact space between the denture and the adhesive and, also, the contact space between the adhesive and the oral mucosa. Both these contact spaces were modelled as bonded contact formulations. The bonded contact formulation restrains the relative displacement at these contact spaces and thereby the partial denture, which resembles the general functioning of an adhesive. The denture clasp-abutment tooth contact spaces were modelled as frictional contacts with a coefficient of 0.3 for both of the models.  Figure 1(a) describes the isometric view of the base model with the adhesive layer implementation between the denture and the oral mucosa.  Figure 1(b) illustrates the side sectional view describing the model in greater detail.

**Figure 2:**
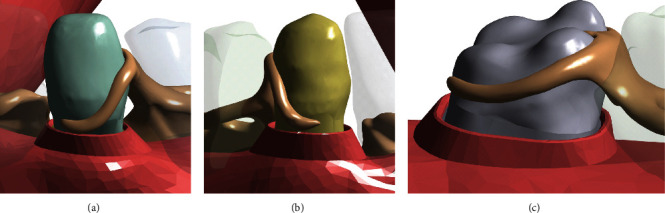


The root geometry of the three molar and canine abutment teeth in the model was used to develop their corresponding periodontal ligament (PDL) geometries. The surface extracted from the root region was used to define a uniform layer with a thickness of 0.2 mm [12, 13]. The alveolar process of the mandible was developed based on the sweep profile created by approximating the surface of the jawbone and the PDL. Boolean unite operations were used to then develop the complete geometry of the oral mucosa. This process was repeated for all of the abutment teeth to model their respective gingival regions. The results are illustrated in Figures 2(a)–2(c) for the left canine, right canine, and molar abutment regions of the oral mucosa, respectively.

The storage modulus (**G**′) and loss modulus (**G**^″^) values from the literature for the adhesive poly(3,4-dimethoxystyrene/styrene-alt-maleic acid) P(DMS/S-alt-MA)2 [5] were used to model the adhesive's behavior. The Prony series approximation [14] method was used to fit the rheological data and, as well as, model it in the FE simulation environment. The adjusted *R*-squared value was used to decide the number of Prony parameters (*N*). Based on this approach, *N* = 4 was used for a corresponding fit accuracy of 99.9%. This iteration was stopped at *N* = 4 as a further increase in the number of parameters produced either a negligible increase in accuracy or a reduction. The mathematical formulation for such a four-parameter Prony series is given in Equation (1).

(1) $$\begin{array}{c} G\left(t\right)=G_{\infty }-\overset{N=4}{\underset{i=1}{\sum }}g_{i}\left(1-e^{t/\tau_{i}}\right) \end{array}$$

Here, **g**_**i**_ is a material constant indicating the stiffness of each branch of the generalized Maxwell model, and **τ**_**i**_ is the corresponding relaxation time. *G*_∞_ is the stiffness of the additional parallel linear elastic spring at infinite time. The shear relaxation function *G*(*t*) was estimated based on shear storage (G′) and shear loss modulus (G^″^) values. The Prony parameters obtained by curve fit based on Equation (1) were used to model the viscoelastic behavior of the adhesive layer in the FE simulation. The 3^rd^ order Ogden model was used to describe the PDLs [15], wherein the material constants, *μ*_*i*_ and *α*_*i*_, were taken from the literature and are illustrated in Table 1 [16]. The abutment teeth, oral mucosa, mandible, partial denture segments, and denture connector were all modelled as isotropic and linear elastic materials based on the literature [17–19]. Table 1 illustrates the material properties used for these components in detail.

The meshes were generated for the models based on a mesh convergence study performed using skewness as the primary mesh metric. The average skewness of the mesh was 0.28, which falls under the “good” criteria of ANSYS WB mesh guidelines. A bite force of 130 N was applied to the 1^st^ molar region of the right denture segment with an inclination of -9° in the frontal plane and -15° in the sagittal plane [9, 20] to study the effect of unilateral biting. Vector components were used to apply this force on the element set consisting of the occlusal surface of the 1^st^ molar region of the right denture segment. The lower surface of the jawbone was applied a fixed boundary constraint by defining an element set. FE simulation was performed for both the base model, i.e., the model without the adhesive layer and the corresponding model with the adhesive layer. The two models were compared with respect to the resultant reaction forces and reaction moments at the six key contact spaces, i.e., the contacts between the denture and oral mucosa as well as the contacts between the denture and the abutment teeth. The share of the transmission of the applied bite force through the soft tissue and the abutment teeth was compared, and the role played by the adhesive was analyzed in influencing the distribution profile across the two models. Further, the effect of the force transmitted through the soft tissue was evaluated by comparing the contact mechanical state of the two models at the denture-oral mucosa contact spaces. Additionally, the von Mises stress levels induced by the share of bite force transferred through the soft tissue was compared for the two models with respect to pressure pain threshold values for soft tissues. The influence of the denture adhesive was observed in these comparisons. Simulations were performed using 10 load steps in 10% increments of the applied bite force.

## 3. Results

The Prony series parameters, amplitudes, **g**_**i**_ and relaxation times, **τ**_**i**_ calculated from the G′ and G″ values [5] are shown in Table 2. Using parameters detailed in Table 2, the adhesive layer was implemented as a viscoelastic material using a Prony series approach. The reaction forces in the oral mucosa due to the applied bite force are described in this section.

**Figure 3:**
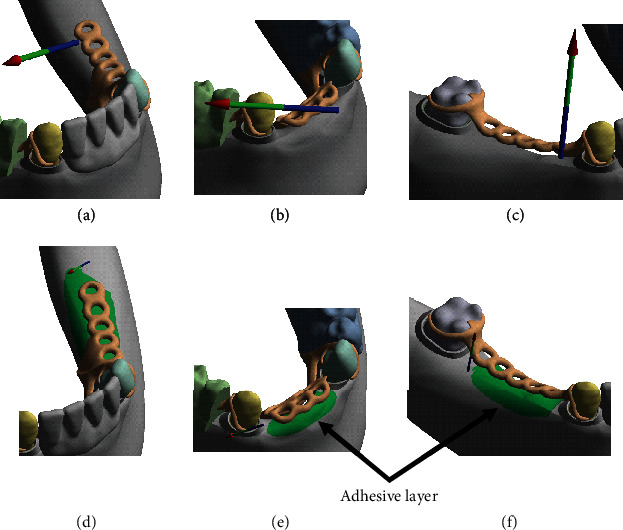


The reaction forces due to the applied bite force on the right denture segment are illustrated in Figure 1 for the models with and without the adhesive layer. Figures 3(a)–3(c) illustrate the resultant reaction forces observed without the implementation of the adhesive layer in the right denture-oral mucosa contact space, center denture-oral mucosa contact space, and the left denture-oral mucosa contact space, respectively.

The absolute value of the resultant reaction force observed for the right denture-oral mucosa contact space was 66.58 N with components -0.97 N, -17.38 N, and -64.26 N along the *x*, *y*, and *z* global coordinates, respectively. The corresponding reaction force with the implementation of the adhesive layer is illustrated in Figures 3(d)–3(f) for the three oral mucosa contact spaces. The corresponding absolute value of the resultant reaction force on the right denture-oral mucosa contact space with the adhesive layer was 30.66 N with components 0.45 N, -5.43 N, and -30.17 N along the *x*, *y*, and *z* global coordinates, respectively. We observed an absolute value of the resultant reaction force of 5.35 N on the center denture-oral mucosa contact space without the adhesive and 2.89 N with the adhesive. Similarly, for the left denture-oral mucosa contact space, a resultant reaction force of 3.15 N was observed without and 0.78 N with the implementation of the adhesive layer. The resultant reaction in Table 3 also clearly shows that with the implementation of the adhesive, the reaction forces on the oral mucosa contact spaces are lowered by approximately 50% for the right and center denture contact spaces and further by approximately 75% in the case of the left denture contact space. On the other hand, the reaction forces for the three abutment tooth contact spaces are higher for the model with the adhesive layer. The maximum reaction forces are observed in the molar abutment clasp contact space due to the proximity of the bite force location. Of particular note are the reaction forces for the left denture segment, as the applied bite force location was on the right denture segment as described in Figure 3. This effect on the left denture-oral mucosa contact space is further highlighted when considering the variation of reaction moments as well.

Table 4 summarizes the resulting reaction moments in *N*-mm seen within the three denture-oral mucosa contact spaces as well as the three abutment teeth contact spaces due to the applied bite force.

The reaction moments also decreased with the implementation of the adhesive layer for the denture-oral mucosa contact spaces and, similar to the case seen for reaction forces, also increased for the denture clasp-abutment tooth contact spaces. In particular, the reaction moments for the left denture segment, which is remote compared to the applied load location, decreased from 43.91 N-mm without the adhesive to 5.78 N-mm with the adhesive. Consequently, the opposing trend was observed for the abutment clasp-teeth contact spaces. The absolute value of the reaction moments increased from 1.9 N-mm for the model without the adhesive to 7.1 N-mm for the model with the adhesive in the case of the left canine abutment clasp contact space. Similarly, the reaction moments also increased for the molar and right canine abutment clasp contact spaces as well.

**Figure 4:**
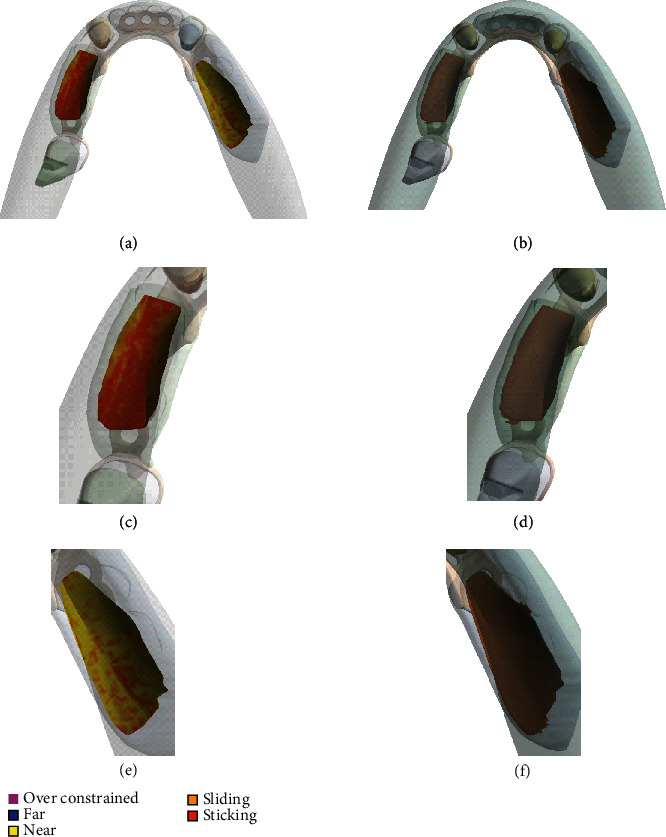


The contact state due to the application of bite force is illustrated in Figures 4(a) and 4(b) for the models with and without the application of the adhesive layer.  Figure 4(c) indicates predominantly a near sliding or sliding contact condition in the left denture-oral mucosa contact space without adhesive, whereas inclusion of the adhesive ensured a sticking condition as illustrated in Figure 4(d).

**Figure 5:**
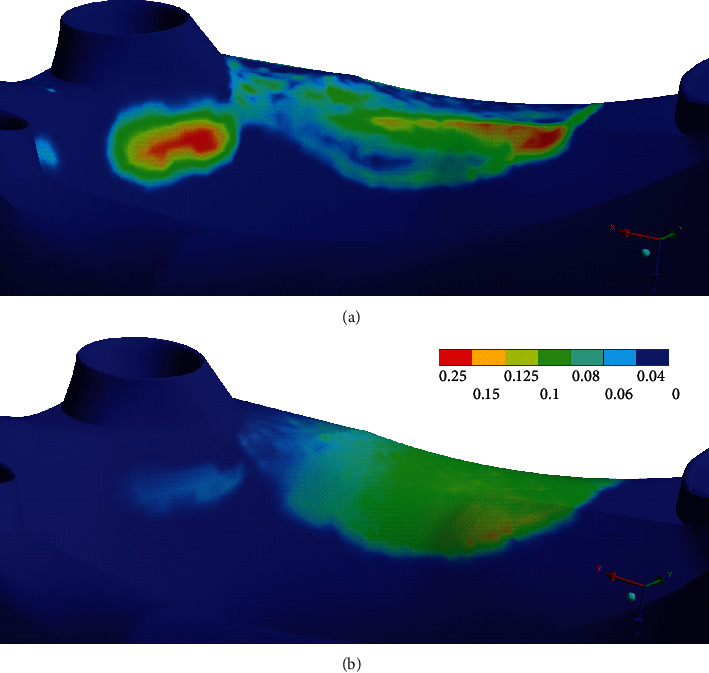


Furthermore, even in the case of the right denture which is directly subjected to the applied force, several distributed regions of a near sliding contact condition are observed, as seen in Figure 4(e). A sticking condition is consistently observed only for the regions of the contact space directly below the applied force. In contrast, the application of adhesive application resulted in a uniform sticking condition across the entire area of the denture-oral mucosa contact spaces as shown in Figures 4(d) and 4(f) for the left denture and right denture contact spaces with respect to the oral mucosa. The contact pressure for the two models with and without the adhesive layer is compared in Figure 5. A maximum contact pressure of 0.25 MPa was observed on the right denture-oral mucosa contact space for the model without the adhesive. The corresponding contact pressure for the model with the implementation of the adhesive layer decreased to 0.15 MPa in the right denture-oral mucosa contact space. The contact pressures for the left and center denture contacts are lower than that of the right denture contact space due to their distance from the point of load application.

## 4. Discussion

**Figure 6:**
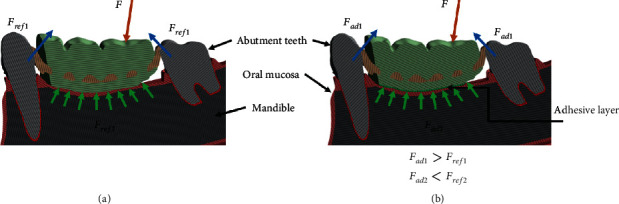


As seen in this study, the stress state of the contact space between the denture and the oral mucosa changes considerably with the implementation of an adhesive layer with a thickness of 0.3 mm. The changes in the resulting reaction forces seen in this study indicate a clear redistribution of stresses between the contact spaces. The bite force, **F** applied on the denture can transfer either through the abutment clasps to the abutment teeth (**F**_1_) or through the soft tissue on which the denture rests to the underlying mandible (**F**_2_). The change in the force distribution is illustrated in Figures 6(a) and 6(b) for the reference model without the adhesive and the model with the adhesive, respectively. For the reference model without the adhesive, larger force transfer is seen through the three denture-oral mucosa contact spaces (**F**_**ref**2_) and relatively lower force through the three abutment teeth-denture clasp contact spaces (**F**_**ref**1_). The friction contact condition implemented in this study for the case without the adhesive allows for the denture to have both translation and rotational movements in the *x* and *y* directions. In the *z* direction, the movement is infinitesimal due to the contact with the mucosa and the underlying bone. For the model with the implementation of the adhesive layer, the applied bite force is again transferred through both the oral mucosa contacts and the abutment contacts. However, the share of the load transferred through the oral mucosa, (**F**_**ad**2_), is lower than (**F**_**ref**2_), while the load transferred through the three denture clasps, (**F**_**ad**1_), is higher than that for the reference model, (**F**_**ref**1_). The bonded contact formulation between the denture and oral mucosa contact spaces restricts both the translational and rotational degrees of freedom of the denture along the *x*, *y*, and *z* global coordinates.

The distribution of higher forces via the denture clasps to the abutment teeth due to the implementation of the adhesive layer can help lower the adverse effect on the soft tissue of the oral mucosa. Figure 5 illustrates this effect on the oral mucosa due to the adhesive layer. The difference in the absolute value of the contact pressures may not be significant, as the values can change with the location and magnitude of the applied bite force as well as the boundary constraints for the model. Qualitatively, however, for a given bite force and point of application, this study shows that the adhesive restrains the denture, and consequently, the force distribution is altered such that the force through the soft tissue is decreased (i.e., **F**_**ad**2_ < **F**_**ref**2_).

On the other hand, the force distribution through the abutments is relatively higher with respect to the model without the adhesive (i.e., **F**_**ad**1_ > **F**_**ref**1_). The lower force on the soft tissue can correspondingly help in keeping the resulting stresses within or significantly lower than the pressure pain threshold for oral mucosa. The contact state in Figure 4 shows the second outcome from this study. The near sliding or sliding contact conditions seen across large sections of the contact space correlate well with the contact pressure described in Figure 5. The near sliding or sliding contact condition seen for the reference model in the case of the left denture segment, which is not loaded in this study, can potentially slide or lift off, which can cause irritation and perceivable pain to the denture wearer. Additionally, the area under the right denture, which is directly loaded, does not show a well-defined stick condition. Regions remote to the bite force are subject to changing contact mechanical criteria, especially the outer extremities of the denture contact region. On the other hand, the stick contact condition throughout the denture-oral mucosa contact spaces for the model with the adhesive restricts liftoff of the left denture as illustrated in Figures 4(d) and 4(f). This reduction in the degrees of freedom for the denture segments can reduce the pain experienced by denture wearers. Figures 4(d) and 4(f) show that the denture remains restricted to the initial contact space before the application of bite force. Hence, a uniform positive contact between the dentures is seen with the adhesive, which is further illustrated in the pressure plot (Figure 5(b)). Not only are the stresses lower than for the reference model without the adhesive, but there is also a uniform distribution throughout the contact space. There are no stress concentrations around the canine abutment tooth and directly around the area of bite application as compared to the model without the adhesive. This denture retention and the corresponding change in the force distribution can be significant when more complex and longitudinal studies are performed.

## 5. Outlook

The in situ response of denture adhesives depends on several additional parameters which could not be factored into this study. Adhesive performance is significantly affected by physical parameters such as temperature, pH, and adhesive swelling. Additionally, in reality, loading scenarios are a combination of both forces (as assumed in this study) as well as moments generated due to the motion of the lower jaw during the process of biting and chewing [21]. The use of point forces or sphere contact to model occlusal loading during mastication overestimates the magnitude of enamel stress and also influences enamel stress distribution [22]. Thus, a comprehensive viscoelastic model of the adhesive that considers the influence of pH, temperature, and swelling ratio coupled with complex loading would improve our understanding of the behavior of adhesives with greater detail.

## 6. Clinical Relevance

There is a qualitative effect on the stress distribution over the soft tissue with the use of the adhesive layer, as a larger share of the bite force is transferred through the abutment teeth and a comparatively lower share through the soft tissue. This in vitro study serves as a proof of concept, illustrating the possible benefit of adhesives in reducing the stress on the mucosal surfaces under the denture surface. However, only longitudinal simulations and/or clinical studies over appropriate time intervals can substantiate these findings with regard to the possible benefits of denture adhesives for patients.

## Figures and Tables

**Table 1:** Material parameters used for the components in the FE model.

	Element	Young's modulus (MPa)	Poisson's ratio	Material model
1	Mandible [17]	1 × 10^4^	0.3	Isotropic, linear elastic
2	Oral mucosa [19]	1 × 10^1^	0.4	Isotropic, linear elastic
3	Abutment teeth [17]	8.4 × 10^4^	0.33	Isotropic, linear elastic
4	Denture segments (PMMA)	1.8 × 10^3^	0.4	Isotropic, linear elastic
5	Denture connector (surgical steel) [17]	1.9 × 10^5^	0.3	Isotropic, linear elastic
6	Abutment periodontal ligaments [16]	**μ**_**i**_	**α**_**i**_	
	**i** = 1	-24.424	1.999	Hyperelastic
	2	15.897	3.999	
	3	8.569	-2.000	

**Table 2:** Parameters of Prony series approximation calculated by Equation (1) based on G′ and G^″^ values for the adhesive cream.

Parameter (*i*)	Relative moduli (**g**_**i**_)	Relaxation time (**τ**_**i**_)	Standard error for fit **g**_**i**_ vs. **τ**_**i**_
1	0.9701	0.0155	1.1E-5
2	0.0053	0.6143	-1.64E-4
3	0.0196	0.0932	1.51E-7
4	3.657E-15	0.7832	4.71E-5

**Table 3:** Summary of the reaction forces on the major contact spaces in the assembly due to the applied bite force on the right denture segment.

	Contact space	Without adhesive				With adhesive			
		Reaction force components along global coordinates (*N*)			Absolute value (*N*)	Reaction force components along global coordinates (*N*)			Absolute value (*N*)
		*x*	*y*	*z*		*x*	*y*	*z*	
1	Right denture-oral mucosa	-0.97	-17.38	-64.26	66.58	-0.97	-17.38	-64.26	30.66
2	Center denture-oral mucosa	-2.78	4.32	-1.51	5.35	-2.78	4.32	-1.51	2.89
3	Left denture-oral mucosa	0.63	2.81	-1.26	3.15	0.63	2.81	-1.26	0.78
4	Abutment clasp-right canine	-9.64	3.48	-8.06	13.04	11.33	0.88	-22.16	24.91
5	Abutment clasp-molar	13.84	6.31	-52.06	54.24	-8.38	2.51	-78.12	78.61
6	Abutment clasp-left canine	-7.58e-2	-9.09e-2	-0.3	0.32	-2.56e-2	0.5	6.11e-3	0.50

**Table 4:** Summary of the reaction moments generated at the major contact spaces in the assembly due to the applied bite force on the right denture segment.

	Contact space	Absolute value of reaction moment (*N*-mm)	
		Without adhesive	With adhesive
1	Right denture-oral mucosa	293.4	57.02
2	Center denture-oral mucosa	10.84	6.93
3	Left denture-oral mucosa	43.91	5.78
4	Abutment clasp-right canine	37.82	75.56
5	Abutment clasp-molar	142.17	273.43
6	Abutment clasp-left canine	1.9	7.1

## Data Availability

Data is available on request.

## Abbreviation

RPD: Removable partial denture.
